# Exploring the impact of providing evidence-based medicine training to service users

**DOI:** 10.1186/s40900-015-0010-y

**Published:** 2015-08-20

**Authors:** Andy Gibson, Kate Boddy, Kath Maguire, Nicky Britten

**Affiliations:** 1 Department of Health and Social Sciences, University of West England, Glenside Campus, Room 2G27, Bristol, UK; 2 NIHR CLAHRC South West Peninsula (PenCLAHRC), University of Exeter, Veysey Building, Salmon Pool Lane, Exeter, EX2 4SG UK

**Keywords:** Evidence-based medicine, User involvement, Public involvement, Training

## Abstract

**Plain English summary:**

Within health services research in the UK, there has been growing interest in evidence-based medicine (EBM) and patient and public involvement (PPI) in research. These two movements have a common goal of improving the quality and transparency of clinical decision making. So far, there has been relatively little discussion about how these two movements might relate to each other, despite their common concern. Indeed, some in the PPI movement have expressed doubts about the implications of EBM for PPI because they worry that its emphasis on evidence from clinical trials marginalises the importance of a patient’s individual experiences in clinical decision making. The purpose of this paper is to examine the potential for EBM and PPI to complement one another.

We analysed the feedback of 10 members of the Peninsula Public Involvement Group (PenPIG) who attended EBM workshops. These workshops trained people in the basics of EBM and were primarily attended by health professionals. We used thematic analysis, a qualitative data analysis method, to explore the responses. We found that participation in the workshops appears to have increased the ability and confidence of members of the public to actively participate as both producers and consumers of research evidence. We conclude that there is an untapped potential for EBM and PPI to complement one another in their shared desire to improve the quality and transparency of clinical decision making.

**Abstract:**

**Background** Within the UK, health services research in the 1990s was marked by growing interest in evidence-based medicine (EBM) and in the potential of patient and public involvement (PPI) in research. However, there has been relatively little discussion of how these two developments might relate to each other, despite their common concern to improve the quality and transparency of clinical decision making. Indeed, some in the user involvement movement have expressed doubts about the implications of EBM for PPI. The purpose of this paper is to examine the potential for EBM and PPI to complement one another.

**Methods**

We used a case study design. Fifteen EBM workshops, involving PPI members, were conducted between June 2010 and December 2014. All 13 lay participants, who attended the first five workshops, were asked to fill in a standard feedback proforma designed by a member of the NIHR Collaboration for Leadership in Applied Health Research and Care for the South West Peninsula (PenCLAHRC) Public Involvement Group (PenPIG). Ten responses were received, and these were analysed thematically.

**Results**

Four themes emerged from the thematic analysis: research knowledge, research skills, shared clinical decision making and learning environment. Participation in the workshops appears to have increased the ability and confidence of members of the public to actively participate as both producers and consumers of research evidence.

**Conclusions**

There is an untapped potential for EBM and PPI to complement one another in their shared desire to improve the quality and transparency of clinical decision making.

## Background

### EBM: definition and origin

David Sackett proposed what is now a widely accepted definition of evidence-based medicine (EBM). “Evidence based medicine is the conscientious, explicit, and judicious use of current best evidence in making decisions about the care of individual patients” [1]. Sackett goes on to elaborate on the three cornerstones of EBM: individual clinical expertise, best available clinical evidence and individual patients’ situations, rights and preferences.

The birth of the modern EBM movement can be traced to Archie Cochrane’s publication of *Effectiveness and Efficiency: Random Reflections on Health Services* in the early 1970s [2]. Here, Cochrane set out his belief that healthcare should be based on treatments shown to be effective by research evidence [3]. Throughout the 1980s, a McMaster University team, including Sackett, worked to develop a practical method to apply the principles of clinical epidemiology to the delivery of healthcare [4]. To develop and research these methods further, the Cochrane Centre was established in the UK in 1992. At the same time, a seminal article on EBM was published in JAMA which described EBM as a new paradigm for medical practice [5]. The first article to describe the steps involved in the method behind EBM was also published in 1992 and was very specific in the advice it gave to clinicians [6]. Since then, EBM has enjoyed a rapid growth of influence, for example, via incorporation into medical curricula, and has been brought to public attention through popular publications such as Ben Goldacre’s *Bad Science* [7].

### Critical view of EBM

Despite this success, EBM has been, from its inception, the subject of heated debate [8]. Critics of EBM have cited numerous limitations. A review undertaken by Cohen et al. categorised these criticisms and limitations into the following five themes: reliance on empiricism, narrow definition of evidence which excludes important information, EBM itself is not evidence-based, limited usefulness for individual patients and threats to the autonomy of the doctor/patient relationship [9]. More extreme criticisms have included describing it as the work of a cult or fascists [10, 11].

### PPI: definition and origin

INVOLVE, the organisation that supports public involvement in the National Health Service (NHS), defines public involvement in research as research being carried out “*with*” or “*by*” members of the public rather than “*to*”, “*about*” or “*for*” them [12]. The 1990s were marked by an increasing interest in patient and public involvement (PPI) within the Department of Health and the NHS [13]. These developments may be seen as a response to three major factors: public response to a series of NHS scandals, public demands for a greater voice in decisions about the services they receive and demands from politicians for greater efficiency and effectiveness in the use of public funds and increased quality of services [14, 15]. The Kennedy report in particular made a strong argument for greater public involvement in the delivery of health services. Although the involvement of service users originated in the provision of services, it has spread into health research, and today, many funders including the National Institute for Health Research (NIHR) require user involvement in grant proposals [16].

However, evidence-based medicine as a concept has largely been ignored by the PPI in the health research community. Those within the PPI in the research community who have engaged with EBM have largely been critical. The common thread of this criticism is that EBM does not account for the individual needs and concerns of the patient. For Faulkner and Thomas, EBM reduces a patient to “little more than a disease being treated” and is ethically problematic [17]. Glasby and Beresford, whilst acknowledging that evidence-based practice is valuable in principle, are very wary of what constitutes “evidence” in EBM. They criticise EBM for its emphasis on the importance of systematic reviews. They state that the dominance of systematic review evidence, which traditionally synthesises randomised controlled trials, marginalises the views and experiences of those who use health services [18].

### The knowledgeable patient: EBM and PPI

Despite these criticisms, EBM has been promoted by some as a vital tool to aid patient empowerment. Bero and Jadad made a case for systematic reviews to inform the decisions made by consumers and policy makers and, as early as 1997, argued that consumers should be involved in the production of reviews [19]. Others have argued that a better understanding of EBM by consumers could promote a more realistic understanding of the effectiveness of treatments [20], raise awareness about variation in practice [21], empower the public in their interactions with healthcare professionals [22] and aid participation in decision making related to research [23]. It has also been suggested that consumer demand for EBM could act as “bottom-up” approach to prompting the adoption of evidence-based medicine by health professionals [20, 24]. Far from marginalising patient views, Domenighetti suggests that EBM, “leads to more autonomy and freedom for the individual”. Sackett, one of the originators of modern EBM practice, goes further, stating that “evidence based patient choice empowers patients and saves their lives” [21].

There are very few reports of approaches and methods of providing EBM to non-health professional audiences. Those few papers that discuss public engagement with EBM generally discuss the concept from a theoretical stance and are vague about the practical details. Suggestions also tend to focus on particular issues, for example, producing leaflets about the efficacy and safety of ultrasonography during pregnancy or mass media campaigns about specific concerns such as prevalence of hysterectomy [19, 20]. This form of engagement with research is passive and instrumental and does not afford an individual the kind of empowerment described by Sackett [21]. The authors found only a few reported attempts to actively engage the public using methods derived from EBM. The first consisted of a set of six questions that patients could ask their doctors about treatment options. These questions were intended to help patients make requests for evidence-based treatments. The authors of that study planned to publish the questions as a booklet and distribute them to households in Switzerland. No follow-up data for this intervention was found [20]. Two reports represent more substantial attempts to provide consumers with the tools to engage with EBM and therefore to obtain some of the benefits of EBM described above. Both reports, from the same research team, describe a detailed curriculum, similar in style to those delivered to health professionals [25, 26]. In one report, the team delivered a 5-day curriculum, called “EBM@school”, to a group of school children aged 16–18 years. In the other, the adapted curriculum was delivered to patient and consumer representatives. Both papers focus on the feasibility of delivering EBM training to non-professional groups and the acquisition of skills and knowledge. More recently, a group in California, USA, report the successful provision of critical skills training workshops to mixed audiences including consumers [27].

These studies reported that teaching EBM skills to these audiences was feasible and enhanced participants’ critical health literacy. Although these reports describe active engagement with the public, it is important to note that it is as *consumers*, rather than *producers* of research.

The Cochrane Collaboration has established the Cochrane Consumer network. Members of the network are asked to appraise Cochrane systematic reviews before publishing. They do this as part of a team which may also include a service provider or researcher in the same area of healthcare as the review, a statistician and members of the editorial board of the Cochrane review group. Team members are given a checklist and prompt sheet and feedback to the review group. This is one of the few examples of active public participation in an EBM-based research process [28].

Given the lack of published reports about EBM and PPI, it is perhaps unsurprising that authors such as Glasby and Beresford have been sceptical about the potential for EBM to empower patients and members of the public. Some may argue that patient empowerment (however defined) is more easily achieved in non-medical environments. The potential dangers of attempting PPI in a medicalised environment include resistance from professionals and the co-option of patients into an agenda which is set by the medical profession.

Others have criticised such initiatives for falling foul of what Ives et al. term the “professionalization paradox” [29]. They suggest that the process of providing EBM-type training undermines precisely that which PPI values most, the ability to bring a lay perspective to bear on research. They argue that providing this type of training necessarily involves some degree of professional socialisation. As a result, they suggest that the ability of the lay person to bring the benefits of “layness” to research is undermined. This is not because members of the public lose their experience of illness, disability or accessing services as a result of taking part in training but because their lay perspective is “tamed” to bring it more in line with that of the professional researcher. This position runs contrary to current advice offered by the UK’s leading involvement organisation, INVOLVE [30]. Indeed, some funding bodies not only require researchers to detail their public involvement plans but also how they intend to support and train those who are involved [31]. The NIHR Collaboration for Leadership in Applied Health Research and Care for the South West Peninsula (PenCLAHRC) regards training for PPI members as a vital part of the involvement process and central to the facilitation of meaningful, as opposed to tokenistic, involvement.

One reason the proponents of EBM for the public talk theoretically rather than from experience may be the practical difficulties involved in delivering EBM to the public. Providing EBM training to health professionals in healthcare settings has been problematic [32], and successful delivery to the public raises different but equally challenging difficulties. Bero and Jadad acknowledge only one main difference between policy makers and consumers when using systematic reviews, that of perspective, seeing policy makers as taking a general population perspective and consumers as concerned with their own perspective [19]. They fail to acknowledge that consumers and policy makers have very real differences when it comes to accessing systematic reviews, understanding and evaluating them and applying the results to their own healthcare decision making. Peninsula CLAHRC with its strong commitment to, and investment in, both EBM and PPI provides a unique opportunity to explore some of these issues in more depth. The aim of this paper is to explore the potential for EBM and PPI to complement one another and the impact of providing EBM training to service users.

## Methods

### The case

We used a case study design. The case was the Peninsula Public Involvement Group (PenPIG) of the Peninsula CLAHRC. PenCLAHRC is a partnership of all the local NHS Trusts across Somerset, Devon and Cornwall, plus the Universities of Exeter and Plymouth. Its objectives are to support the identification of research questions that address clinical and patient concerns, to support and undertake research that test treatments and new ways of working in specific clinical areas and, where effective interventions are identified, to support research into how NHS staff can implement them into everyday working practices. It is part of the Collaboration for Leadership in Applied Health Research and Care (CLAHRC) programme which arose from the Chief Medical Officer’s High Level Enquiry into Clinical Effectiveness. This enquiry was asked to consider why there was variation in clinical practice and what the NHS should do about it [33]. The report concluded that unacceptable variations in clinical practice are in part driven by (a) the lack of high-quality evidence addressing many key clinical questions, combined with (b) the continuing failure to implement much existing evidence. Recommendations included developing new frameworks for closer working between clinicians, managers in the health and social care services and academics in higher education.

In response, the NIHR funded regional partnerships between universities and NHS organisations to establish pilot CLAHRCs. These partnerships are designed to undertake high-quality applied health research focussed on the needs of patients and to support the translation of research evidence into practice in the NHS.

One of the distinctive elements of PenCLAHRC’s approach has been the emphasis that it has placed on the importance of PPI in research. This is partly based on an ethically driven position that the public have a right to influence research priorities. It is also based on the view that patients are the final decision makers in the chain of implementing research evidence. Ensuring that research deals explicitly with patients’ information needs and perceptions of the issues is therefore likely to increase the utility of research findings, thus increasing the probability of effective use of evidence in practice. Achieving this requires the development of effective methods for the engagement of members of the public both in helping to set the research agenda and in shaping specific projects.

To help achieve these aims, PenCLAHRC has set up the PenPIG. PenPIG is a user-led advisory group made up of members of the public, service users and carers. At the time of writing, there are 14 members drawn from a wide geographical area in the UK’s South West. PenPIG advises PenCLAHRC on all aspects of user involvement and acts as a “critical friend”. This group also has representation on PenCLAHRC’s Management Board. Members of PenPIG are involved in a wide range of activities including research prioritisation, co-writing applications for research funding, advising and supporting user involvement in ongoing research projects and presenting at conferences [34].

### EBM workshops

PenCLAHRC’s 1-day “Making Sense of Evidence” workshop is designed to be an introduction to EBM [35]. It is based on the principles promoted by Oxford University’s Centre of Evidence-Based Medicine (CEBM) [36]. The workshop programme covers the key elements of EBM (see Tables 1 and 2). A workshop format is used as it allows for an interactive learning environment which includes didactic presentations and small group activities to consolidate learning and build confidence. The course materials and programme are available online [35].

**Table 1:** Introduction to the EBM workshop programme

Session	Content
9.15 Registration and coffee	
9.30 Welcome and introductions	Introduction to the concept of EBM
10.00 Formulating a focused question	Focus recent clinical questions using the PICO formula.
10.45 Tea, coffee and networking	
11.00 Tracking down the evidence	Use key online resources to locate evidence
	Apply PICO to search techniques
12.30 Lunch (and networking)	
1.30 Critical appraisal of an RCT	Overview of the critical appraisal purpose and process
	Experience of using a critical appraisal tool
3.00 Tea, coffee and networking	
3.15 Introduction to systematic reviews and application of evidence paper	Introduction to systematic review process
	Experience of critically appraising a review
4.00 Where do I go next?	
4.30 Close

**Table 2:** Evidence-based practice workshop course objectives

Course objectives
At the end of the course participants will have
1. The ability to format focused questions
2. The ability to identify the best evidence by using search skills and organisation of evidence
3. Experience of critically appraising at least one study type
4. Introductory understanding of the application of evidence to practice
5. Introductory understanding of the synthesis of evidence
6. Ability to know where to go next/make use of resources provided by this workshop

All tutors on the workshop have attended the 5-day workshop “Teaching Evidence-Based Practice” delivered by CEBM. The workshops are primarily aimed at health professionals and attract a range of participants from a variety of specialities and disciplines including, for example, midwives, nurses, surgeons, GPs, allied health professionals and public health consultants. The workshops receive excellent feedback. Typically, each workshop’s content and presentations receive an average score of 4.59, using a 5-point scale where 1 is very poor and 5 is very good. There is also an emphasis on putting the skills acquired into practice in the workplace and a recognition of the challenges of creating a sustained evidence-based practice, details of which have been reported elsewhere [37].

The EBM workshops were initially offered to all members of PenPIG and subsequently to all PPI members involved in specific research projects as well as PPI members of other local involvement groups. The decision to include members of the public in these workshops was made for two reasons. One was that many members of the public who we have worked with had expressed an interest in finding out more about health service research. The other was that the PPI researchers (AG and KB) felt that raising awareness of EBM would improve people’s ability to actively contribute to PenCLAHRC’s work. Four places (out of a total of 24) at each workshop are reserved for PPI members.

### Preparation for involving the public in the workshops

To support and facilitate PPI attendance at the workshops, a number of steps were taken. A PPI member, who had extensive experience of involvement in research, attended a workshop, along with one of the authors (AG), to assess their suitability as a learning environment for all PPI members, irrespective of experience. They found that the workshops were suitable and were positive about the benefit that attendance could bring. They made a number of recommendations to the workshop organisers aimed at ensuring PPI members could attend and fully participate in the workshops. These recommendations, which were all implemented, included the creation of a jargon buster to explain some of the terminology used; a short pre-workshop meeting of about 1 h, solely for PPI members, conducted a week or so before the main workshop to provide an overview of the day and an explanation of the main concepts; and a PPI facilitator to attend on the day to provide extra support if required during the teaching sessions. As far as the authors are aware, this is the first report of EBM workshops delivered to mixed groups of both clinicians and PPI members within the UK.

At the time of writing, 15 one-day workshops have been conducted between June 2010 and December 2014. The 2015 programme is currently underway. The first 13 lay participants of the workshops were members of PenPIG. Their participation provided us with the opportunity to conduct a case study of the impact of providing training in EBM to members of a public involvement group within an organisation undertaking applied health research. We did not seek ethical approval for this case study because guidance published by the NHS National Patient Safety Agency National Research Ethics Service (NRES) and INVOLVE in January 2009 states that ethical approval is not required for patient and public involvement activities that do not include direct contact with study participants [38]. All those who gave feedback on the workshops described in this paper gave permission for their material to be used for publication.

The 13 participants were asked to fill in a standard proforma (see Table 3). This proforma was developed by another member of PenCLAHRC’s public involvement group (KM). The anonymised proforma transcripts were analysed thematically [39]. The data were analysed independently by AG and KB and then discussed in a data meeting to create an agreed collective analysis.

**Table 3:** Proforma Questions

Proforma Questions
● Why did you get involved in health research?
● Which workshops have you attended?
● Was learning about EBM useful to you?
● Have you been able to use what you have learned?
● If you haven’t been able to use what you have learned please tell us about this and what else did you get out of the experience?

## Results

Of the 13 attendees, 10 responded. The responders included people with diabetes (three), stroke survivors (two), people with experience of mental health problems (four) and one carer. The three non-responders did not possess any particular trait that would differentiate them from the responder group. The four themes that emerged from the thematic analysis were as follows: research knowledge, research skills, shared clinical decision making and learning environment.

### Research knowledge

Unsurprisingly, the acquisition of knowledge about research figured prominently in the responses:

“It has given me an insight into how research proceeds and results are interpreted, and the basics about how statistical reviews of evidence are attempted.” ID 10

Significantly, this understanding extended beyond a developing a better grasp of research methodologies and the interpretation of results to gaining an insight into some of the practical difficulties involved in doing applied health research:

“Have learnt that research often takes much longer than is possible in NHS timeframes of change or requires huge cohorts to prove anything with sufficient evidence to be taken seriously.” ID 6

### Research skills

The acquisition of EBM skills was a prominent feature of the feedback:

“The PICO [Population, Intervention, Comparison, Outcome] process was also useful to help me appreciate how good medical research projects should be formulated and proposed, and how to understand what relevant information to look for in a research project paper or publication, including meta-studies, and where to look for these.” ID 10

Importantly, most of the respondents reported that they had been able to put their newly acquired skills into practice in a number of ways. Many had found that it helped them to carry out their role as lay people involved in research:

“I am more able to accommodate and assimilate examining research; the most recent example is a request to lay review a bid.” ID 3

The acquisition of these skills also appears to have improved people’s confidence to participate within academic research:

“Every workshop I have attended has helped to increase my confidence in formulating questions, seeking answers and critically assessing the evidence I find. I was quite shocked to find out how little practice is based on evidence, but felt very empowered by being given access to the skills that could help me to better appraise this for myself.” ID 1

Acquiring these skills also appears to have had a more general impact on the confidence of participants:

“If I must express this succinctly, then I would say that my self-confidence has increased, which is a major step for me.” ID 7

### Shared clinical decision making

Significantly, the reported application of these skills went beyond the academic arena, spilling into people’s interactions with healthcare professionals:

“I use it to research health questions for my own interest, e.g., for breast cancer, and to guide me in discussions with GP and consultants.” ID 2

and:

“One of the elements that was most useful personally was the explanation of some of the statistical data that is commonly presented in news reports. I had often felt that this could be misleading, but the EBP workshops helped me to see how it worked and what I could look for to get a better understanding of what is implied by the numbers, for instance looking at Number Needed to Treat and absolute rather than relative risk.” ID 1

For some, this extended beyond their own personal healthcare to using their skills to influence service improvement:

“I’ve used it quite a bit within mental health settings to help with service improvements in the teams I use and on the wards I visit.” ID 6

Importantly, these experiences seem to have stimulated a thirst for further learning:

“I realise how much more I need (and WANT) to learn.” ID 9

### Learning environment

Participants also fed back on what they felt made a successful learning environment. People valued the opportunity provided in the workshop to practice new skills:

“It was so clearly taught and we could practice during the session.” ID 6

Equally important was the fact that the sessions were enjoyable:

“They were good fun and made me more aware of the sheer breadth of public involvement across the medical spectrum generally.” ID 10

The interdisciplinary nature of the workshops was also important:

“Met lots of interesting people from different professions and back-grounds, and hopefully did a little to break down the barriers that seem to exist sometimes between professionals and lay people, and some of the misconceptions around who takes part in health research-‘The Usual Suspects’.” ID 2

The egalitarian ethos of the groups was also identified as significant:

“A group of friends and colleagues who treat you as an equal no matter what status they have, whether it be Professor, Consultant or Doctor they value the knowledge that you have on the health issues that you suffer from.” ID 1

This comment referring to “a group of friends” also suggests that having several, usually three or four, as opposed to an individual member of the public participating was important in providing peer support and a sense of group learning. This was built on by the provision of opportunities to consolidate the learning and to build confidence through discussions and conversations with other public participants, either in person or by email, along with the provision of a “refresher” session where questions that persisted or were raised later could be addressed.

The centrality of maintaining an informal and friendly atmosphere was also highlighted:

“Gaining knowledge from......is made so easy for us as everyone can ask questions without being made to feel stupid and we are certainly not patronised or made to feel that our contributions aren’t worthwhile.” ID 7

There were few negative comments. Where they occurred, they were related to the practicalities of running the workshops:

“Timing of each session of the workshop needs to be improved-kept over-running.” ID 2

In summary, participation in the workshops appears to have increased the ability and confidence of members of the public to actively participate in the research process. An unanticipated but welcome outcome has been that it has also facilitated some to take a more active role in the management of their own conditions by being enabled to apply relevant research findings to their own situation. Our analysis of the data did not reveal evidence of “professionalisation”, at least in the negative sense as described by Ives et al. of undermining the ability of members of the public to represent a lay perspective [29].

## Discussion

This paper has described some of the impacts of delivering EBM training to service users who are involved in health research. One criticism that could be made of our work is that it involves small numbers and the people involved may not be representative of the wider public. The service users who attended the workshops were interested in health research and had some familiarity with the broad concepts of evidence-based medicine. However, the feedback we received from the service user participants was that these ideas and tools would be of interest and benefit to a general audience of people who were concerned with health issues. We have developed an additional workshop, based on the principles of EBM, and using tools derived from PenCLAHRC’s “Making Sense of Evidence” workshops, designed for a public audience with no prior familiarity with research. These workshops have been delivered to a variety of audiences and have been well received, suggesting that the approach we have described above, with appropriate amendments to suit specific audiences and contexts, has a much wider applicability [40].

## Conclusions

We began this paper by suggesting that there is an untapped potential for EBM and PPI to complement one another in their shared desire to improve the quality and transparency of clinical decision making. Although the case study presented here is on a small scale, we suggest that it provides grounds to support this view. Importantly, the provision of EBM training to members of the public has benefits for both lay and academic participants in the research process. From the perspective of academics, the provision of such training provides members of the public with a knowledge and understanding of the research process that enables them to participate on a more equal footing, thus maximising the utility and impact of their contributions. From the perspective of the public, it increases confidence, is of intrinsic interest and may enable them to take a more active role in the management of their own conditions as “knowledgeable patients” [41]. Giving patients the tools to manage their own conditions allows them to use these tools to pursue their own objectives in ways unanticipated by educators and health professionals.

Furthermore, this paper provides us with a better understanding of the factors and conditions that are necessary for this exchange to happen successfully. It is clear that participants valued practical, hands-on approach, combined with an informal, open and egalitarian learning environment. The techniques described in this paper provide an insight into how we might promote the development of a constructive dialogue between academics and people who have a specific interest in a particular health issue or more widely within the general public.
